# Subjects with Knee Osteoarthritis Exhibit Widespread Hyperalgesia to Pressure and Cold

**DOI:** 10.1371/journal.pone.0147526

**Published:** 2016-01-25

**Authors:** Penny Moss, Emma Knight, Anthony Wright

**Affiliations:** School of Physiotherapy and Exercise Science, Curtin University of Technology, Perth, Western Australia, Australia; Center for Rheumatic Diseases, INDIA

## Abstract

Hyperalgesia to mechanical and thermal stimuli are characteristics of a range of disorders such as tennis elbow, whiplash and fibromyalgia. This study evaluated the presence of local and widespread mechanical and thermal hyperalgesia in individuals with knee osteoarthritis, compared to healthy control subjects. Twenty-three subjects with knee osteoarthritis and 23 healthy controls, matched for age, gender and body mass index, were recruited for the study. Volunteers with any additional chronic pain conditions were excluded. Pain thresholds to pressure, cold and heat were tested at the knee, ipsilateral heel and ipsilateral elbow, in randomized order, using standardised methodology. Significant between-groups differences for pressure pain and cold pain thresholds were found with osteoarthritic subjects demonstrating significantly increased sensitivity to both pressure (p = .018) and cold (p = .003) stimuli, compared with controls. A similar pattern of results extended to the pain-free ipsilateral ankle and elbow indicating widespread pressure and cold hyperalgesia. No significant differences were found between groups for heat pain threshold, although correlations showed that subjects with greater sensitivity to pressure pain were also likely to be more sensitive to both cold pain and heat pain. This study found widespread elevated pain thresholds in subjects with painful knee osteoarthritis, suggesting that altered nociceptive system processing may play a role in ongoing arthritic pain for some patients.

## Introduction

Studies utilizing quantitative sensory testing (QST) data suggest that widespread pressure and cold hyperalgesia are also present in a number of musculoskeletal pain disorders such as tennis elbow [1–3], back pain [4, 5], fibromyalgia [6, 7] and whiplash associated disorder (WAD) [8, 9]. It has been suggested that there is an association between pain severity and chronicity, and the presence of cold hyperalgesia in the immediate period post whiplash injury [10] and based on the findings of a systematic review, the presence of cold hyperalgesia has been identified as an important prognostic factor for long term pain and disability in WAD [11] and tennis elbow [12]. The importance of cold hyperalgesia as a prognostic indicator in osteoarthritis (OA) has not been extensively explored.

Osteoarthritis is one of the most prevalent musculoskeletal disorders affecting Western society and is associated with joint pain, tenderness and decreased function. It is also often anecdotally associated with exacerbations during cold weather conditions. Over recent years QST methods have been used to evaluate various aspects of hyperalgesia in this population [13–20]. Most commonly, studies have evaluated pressure pain thresholds and reported widespread mechanical hyperalgesia in subjects with OA of the knee [16, 20]. Several studies have reported that mechanical hyperalgesia extends beyond the vicinity of the OA joint indicating relatively widespread changes in nociceptive system function. Imamura et al [14] reported reduced pressure pain threshold (PPT) and consequent pressure hyperalgesia at a number of lower extremity sites in subjects with knee OA, correlating with higher disability scores. A number of studies have also reported reduced PPT in the upper limb of subjects with knee OA compared with matched controls [13–15].

It has been hypothesised that this widespread mechanical hyperalgesia may be a sign of altered nociceptive system function and reflects centrally augmented nociceptive system processing [21]. Thus it has been proposed that even in an apparently localised musculoskeletal condition such as OA there may be significant central augmentation of nociceptive input [22, 23]. This hypothesis is also supported by studies that have reported changes in other centrally mediated pain phenomena in subjects with OA. Bajaj at al [24] reported that both the area and intensity of secondary hyperalgesia were increased in subjects with knee OA, following hypertonic saline injection into the tibialis anterior muscle. Temporal summation is also significantly facilitated in this patient group [15, 22]. Studies have found conditioned pain modulation processes to be significantly reduced in patients with OA compared with normals [25].

The presence of cold hyperalgesia has also been proposed as a sign of centrally-augmented nociceptive system processing [21,26]. Whilst animal models of arthritis have demonstrated increased cold hyperalgesia, there are few human studies that have investigated the presence of cold hyperalgesia in patients with OA. Wylde et al [13] carried out a comprehensive evaluation of QST measures in patients with knee OA compared to matched controls. The study demonstrated widespread pressure hyperalgesia and showed no difference in heat pain thresholds but it failed to specifically evaluate cold pain thresholds leaving an important gap in our knowledge base.

Given the potential prognostic importance of evaluating cold hyperalgesia, the current study aimed to investigate the extent to which widespread pressure and cold hyperalgesia is experienced by subjects with knee OA compared to matched, healthy controls. The study also evaluated the presence of heat hyperalgesia and explored associations between cold pain thresholds, pressure pain thresholds, heat pain thresholds and self-report of pain and disability.

## Methods

### Study Design

A cross-sectional case-control design was used. The pressure and thermal pain thresholds of subjects with symptomatic knee OA were compared with those of a healthy, pain-free group, matched by gender, age and BMI. Thresholds were tested in randomised order at three standardised body sites, index knee, ipsilateral heel and ipsilateral elbow.

### Subjects

All subjects were volunteers recruited from the general population using fliers and radio advertisements. Those in the OA group fulfilled the American College of Rheumatology clinical criteria for painful knee OA [27]. Volunteers with any history of chronic pain, fibromyalgia, neurological deficit or co-existing systemic disorder were excluded from both the OA and healthy control groups. Controls were healthy, currently pain-free individuals with no history of knee pain or OA. Controls were matched to OA subjects by gender, 5-year age band and BMI group (normal, overweight, obese). All volunteers provided written informed consent before participating in the study, which was approved by the Curtin University Human Research Ethics Committee (Approval Number: PHTY 42006).

### Procedure

Subjects attended a single one hour test session in a temperature-controlled laboratory maintained at 24°C. Following clinical examination for intact sensory and pain pathways (warm/cool, light touch and pin-prick), all subjects were asked to provide basic demographic data (age, gender, height, weight for BMI) and asked about presence and intensity (numerical rating scale 0–10) of low back pain. All subjects were also asked to detail their average weekly exercise in hours (examples provided such as lawn bowls, walking, cycling, dancing) and then completed the Spielberger State-Trait Anxiety Index (STAI) questionnaire. The OA group also completing the Western Ontario and McMaster (WOMAC) Knee-specific questionnaire. Pain thresholds for pressure (PPT), cold (CPT) and heat (HPT) were tested at three test sites (index knee, ipsilateral heel and elbow) using standardised procedures and scripted instructions. Knee measurements were performed over the medial collateral ligament at the joint line [28], elbow measures were performed over the extensor carpi radialis brevis muscle [29] and heel measurements were performed over the lateral aspect of the calcaneum [30]. For each testing modality at each site, an initial practice was followed by three recorded trials, with the mean values used for analysis. Test order was randomised between subjects for test site and stimulus modality.

### Pain Threshold Measures

#### Pressure Pain Threshold

Pressure pain threshold (PPT), was assessed using an electronic digital pressure algometer (Somedic AB, Sweden), a device that has consistently shown good reliability when applied by a skilled operator [30]. A 1cm^2^ algometer probe was applied at 90° to the skin at a rate of 40kPa/sec. Subjects were instructed to depress the hand-held switch as soon as the sensation of pressure became one of painful pressure. This pressure reading (kPa) was recorded.

In order to evaluate intra-tester reliability for algometry, a small pilot study was completed prior to testing for the current study. Ten subjects of convenience aged between 19 and 30 (mean 21.7 years) were tested using the protocol for PPT described above. Only the knee site was used (medial collateral ligament at the joint line). Subjects were given an initial practice followed by three trails, the mean of which was used for analysis. Subjects were tested on two occasions, separated by 24 hours. Analysis showed an intra-tester Intra-Class Correlation Coefficient (ICC_1,2_) of *r =* .93.

#### Cold Pain Threshold

Cold pain threshold (CPT) was assessed using a peltier thermode (Somedic AB, Sweden) and standard method of limits [31]. The 3x2cm probe was secured to each test site using light strapping to ensure even skin contact. Subjects were given several minutes to adapt to the baseline temperature of 32°C, before the device was activated. The thermode cooled at a rate of 1°/sec down to the minimum available temperature of 5°C. Subjects were instructed to depress the hand-held control switch once the cooling sensation became one of painful cold, thereby reversing the temperature back to baseline.

#### Heat Pain Threshold

Heat pain threshold (HPT) was similarly assessed using the Somedic peltier thermode and Method of Limits described above. Testing started from a baseline temperature of 32°C, increasing a 1°/sec to a maximum temperature of 50°C. Subjects were instructed to press the control switch when the sensation of warmth became one of painful heat.

### Additional Measures

#### Spielberger State-Trait Anxiety Inventory (STAI)

The STAI was used to assess all subjects’ levels of general and situational anxiety on the day of testing. This 40 item self-report questionnaire provides a measure of both state and trait anxiety [32,33]. The STAI is widely used and has demonstrated good reliability (ICC *r =* .65 to *r =* .86) and validity in the context of experimental pain studies [33].

#### Western Ontario and McMaster University Osteoarthritis Index for the Knee (WOMAC)

This self-report questionnaire is widely used to measure pain and disability from knee OA, demonstrating good internal validity and test-retest reliability [34]. The 24 item questionnaire provides an evaluation of three variables; pain, stiffness and physical function, which can be reported separately or as a cumulative score [35].

### Data Analysis

SPSS (v19) statistical package was used, with the α-level set at 0.05. Sample size was calculated using PPT and CPT data from previous studies. Using data from Suokas et al., 2012 [16] a mean difference in PPT between OA and controls across a range of joints of 155.93kPa (standard deviation (SD) 150.29kPa), required a sample size of 15 per group (α-level = 0.05 and β = 0.80). Knee joint PPT data from our own study (awaiting publication) showed that 22 subjects would be needed per group (mean difference 371.18, SD 150.28). Given the lack of available CPT data, our own data was used: a mean CPT difference between OA and controls of 4.68°C (SD 6.99°C) resulted in a sample of 15 per group. It was therefore decided that 20–25 subjects per group would provide adequate power.

Independent t-tests were applied to analyse between-group differences in PPT, CPT and HPT at each test site. In order to analyse responses across modalities, a global value for each modality was calculated as the mean of all 3 sites. These values were also analysed using independent t-tests. Pearson’s Correlation Coefficients were applied to analyse possible associations between the various measures.

## Results

Twenty-three subjects were recruited to the group with knee OA and subsequently matched with 23 healthy control subjects (Table 1). Proportions of male to female subjects in each group (10 male: 13 female) were exactly replicated and there was no significant group difference in mean ages (OA group 68.5 ± 8.5 years: range 55–82 years, control group 66 ± 11 years: range 50–84 years). Mean BMI values for both OA (26.94 ± 4.51) and control groups (25.61 ± 4.10) were in the overweight range, but this reflects current Australian statistics [36]. Both groups were asked to estimate the average time they spent on specific exercise each week (such as walking, bowls, cycling). For both OA and control groups this figure was relatively low at just under 4 hours per week and was not significantly different between groups (Table 1). There were similar numbers of subjects in each group who reported regular low levels of low back pain: 12 OA subjects reported an average intensity of 2.75/10; 11 control subjects reported an average intensity of 2.50/10.

**Table 1:** OA and Control groups: gender, age, BMI, average activity level and anxiety score comparisons.

			*OA Group*	*Control Group*
**Gender** (n): male: female			10: 13	10: 13
**Age** (yrs): mean ± SD^**a**^ (range)			68.5 ± 8.5 (55–82)	66.0 ± 11.1 (50–84)
**BMI**^**b**^: mean (± SD)			26.94 ± 4.51	25.61 ± 4.10
**Activity Level:** (hours/week): mean ± SD			3.78 ± 2.42	3.96 ± 2.76
**STAI^**c**^:** Total score: Score Toy		mean ±SD	66.52 ± 12.70	55.13 ± 9.44
	State Anxiety:	mean ±SD	32.59 ± 9.04	24.95 ± 4.65
	Trait Anxiety:	mean ±SD	34.27 ± 6.34	30.50 ± 7.23

^**a**^ SD: Standard Deviation

^**b**^ BMI: Body Mass Index

^**c**^ Spielberger State-Trait Anxiety Index

OA participants reported a mean symptom duration of 8.26 years (range 2–10 years). WOMAC Knee Index scores for the OA group ranged from 7–70/100 with a mean of 39/100, reflecting a community-dwelling, ambulant cohort with mild to moderate disability. WOMAC subscores showed that this cohort was more limited by stiffness than pain (46% versus 38%) although there was a significant positive correlation between stiffness and pain (p< .001, r = .853) (Table 2).

**Table 2:** Mean (±SD) duration of self-reported symptoms and Western Ontario and McMaster (WOMAC) scores for knee OA subjects.

		*OA Subjects*
**Symptom Duration** (yrs)		8.26 ± 6.17 (2–10)
**WOMAC:**	Pain:	7.68 ± 3.40
	Stiffness:	3.64 ± 1.43
	Function:	27.05 ± 14.44

There were differences in state anxiety (t = 3.52, p = 0.001) and overall anxiety (t = 3.45, p = 0.001) between the OA and control groups (Table 1). The OA group also exhibited higher trait anxiety scores but this difference was not statistically significant (t = 1.839, p = 0.073). No correlations were found between STAI scores and WOMAC pain or function subscores (STAI: WOMAC pain p = .144, r = .523; STAI: WOMAC function p = .224, r = .316). Nor were any correlations found between global pain thresholds and STAI scores (STAI: PPT p = .146, r = .313; STAI: CPT p = .684, r = .090; STAI: HPT p = -.659, r = -.097).

### Pressure hyperalgesia

**Fig 1:**
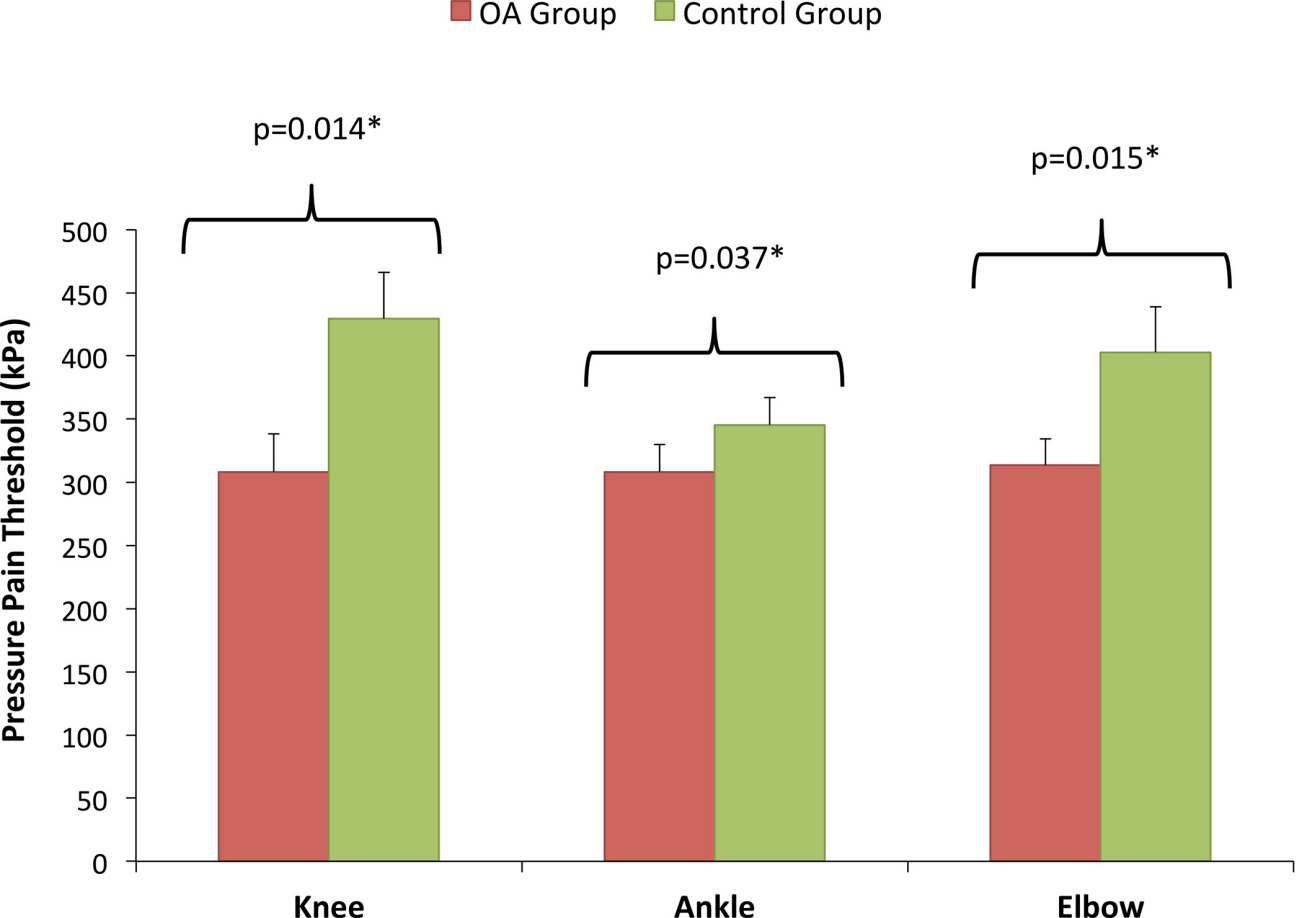
Pressure Pain Threshold. There was a significant difference between OA and Control groups in pressure pain thresholds at both local and distant sites.

Subjects with knee OA exhibited significantly reduced pressure pain thresholds compared with matched controls, both at the index knee (t = -2.57, p = .014) and also at the ipsilateral elbow (t = -2.15, p = .037), and the ipsilateral heel (t = -2.25, p = 0.015, p = .231) (Fig 1). Global PPT values were significantly reduced for the subjects with OA compared to controls (t = -2.44, p = 0.019).

### Cold hyperalgesia

**Fig 2:**
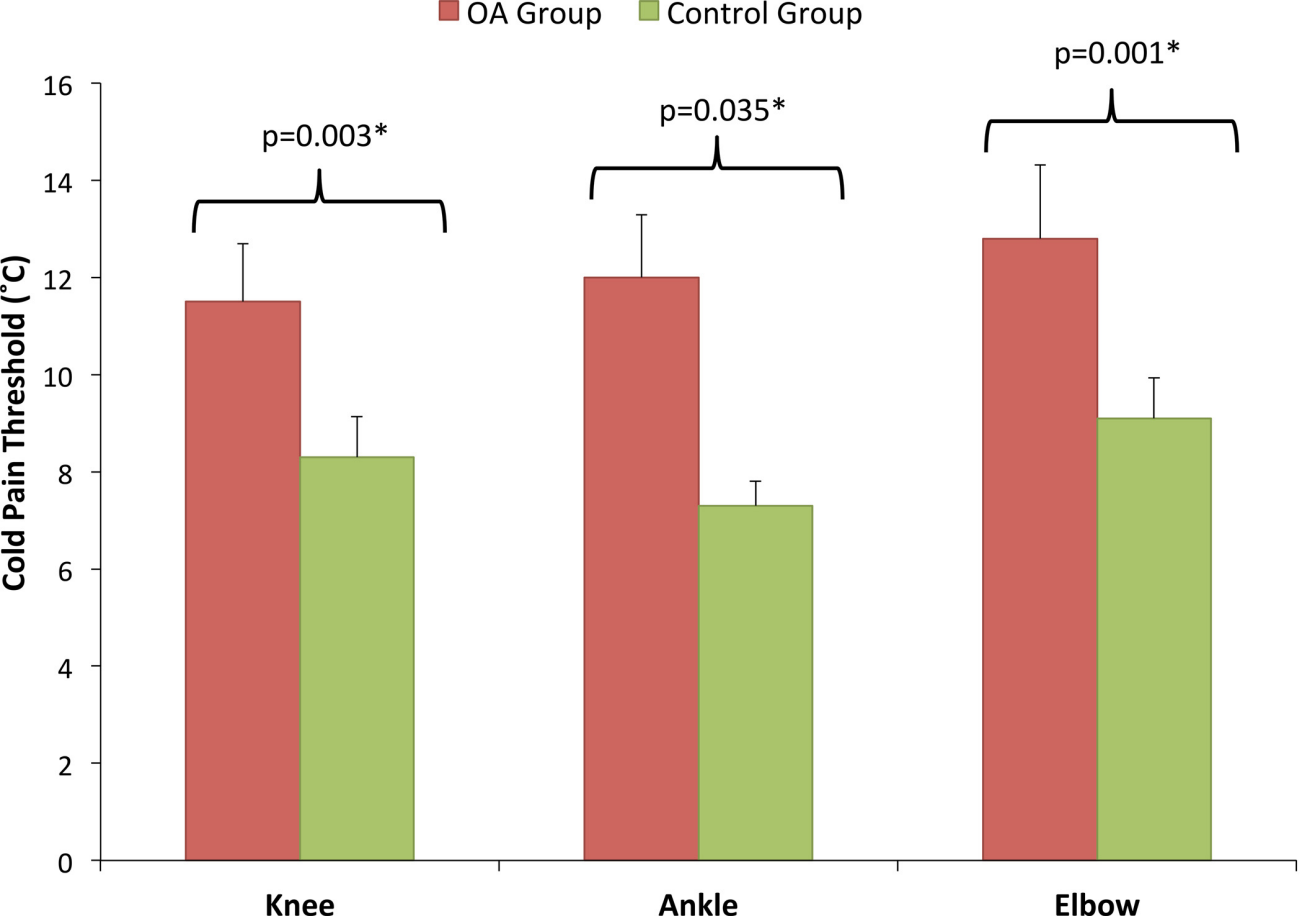
Cold Pain Threshold. There was a significant difference between OA and Control groups in cold pain thresholds at both local and distant sites.

Subjects with knee OA also exhibited significantly higher cold pain thresholds compared with matched controls, at the index knee (t = 2.25, p = 0.03) and also at the ipsilateral elbow (t = 2.18, p = 0.035) and the ipsilateral heel (t = 3.47, p = 0.001) (Fig 2). Global CPT values were significantly higher in the subjects with OA compared to the controls (t = 3.26, p = 0.002).

### Heat hyperalgesia

**Fig 3:**
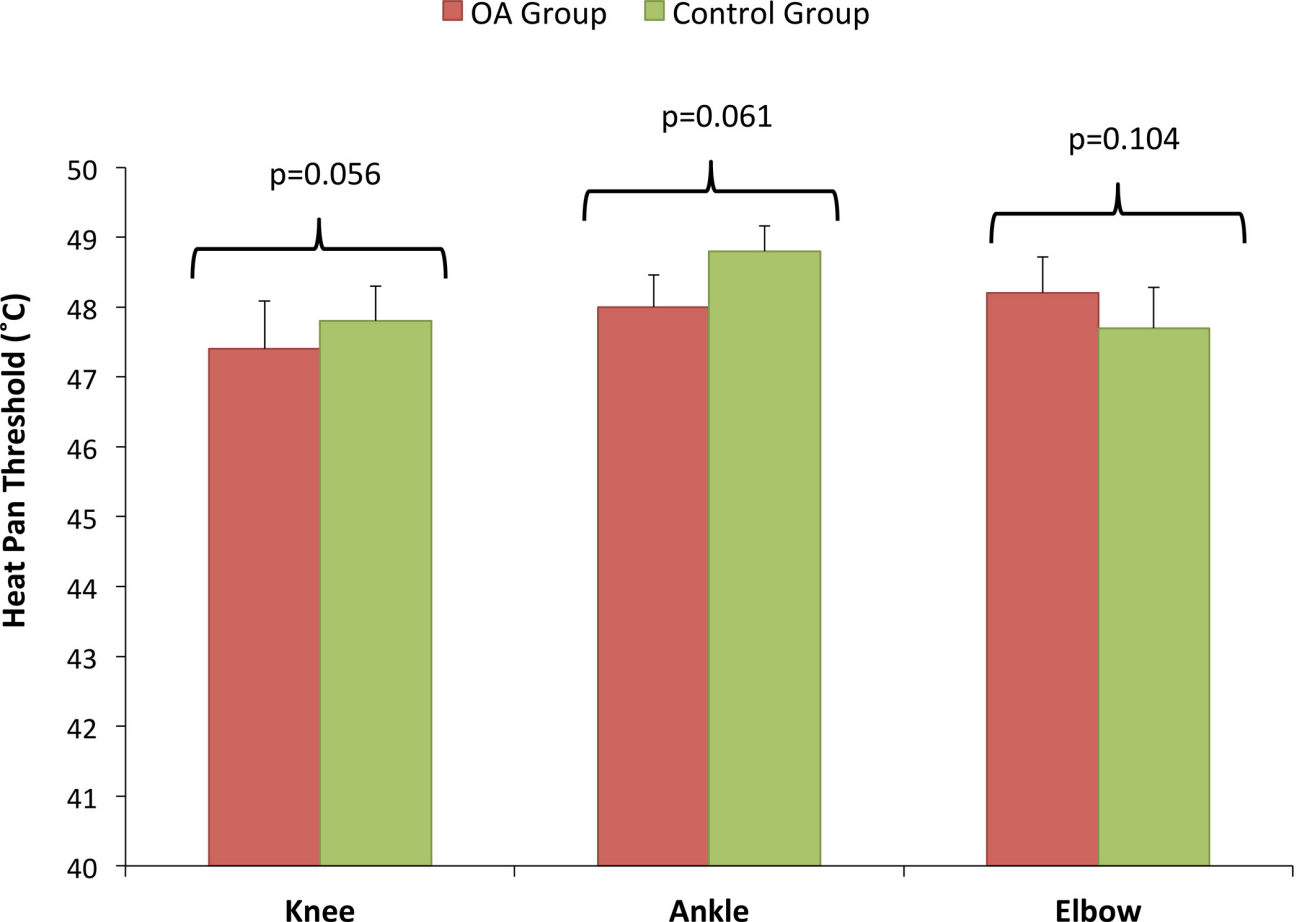
Heat Pain Threshold. There were no significant differences between OA and Control groups in heat pain thresholds.

There were no significant differences in heat pain thresholds at the index knee (t = -0.586, p = 0.56), ipsilateral elbow (t = 0.517, p = 0.61) or ipsilateral heel (t = -0.467, p = 0.104) (Fig 3). There was also no difference in global HPT values (t = -0.584, p = 0.56). A post hoc power analysis indicated that the study had power of 1-β = 0.475 to detect a difference in HPT of 2°C between groups suggesting that an increased number of subjects would be required to detect a difference in HPT should such a difference exist.

### Correlations between modalities

Significant correlations were found between global CPT and HPT (r = -.512, p < .001) as well as between CPT and PPT (r = -.499, p < .001) and HPT and PPT (r = .538, p< .001). Thus subjects with greater sensitivity to pressure pain, were also likely to be more sensitive to cold pain and heat pain. There were no significant correlations between global PPT (r = 0.253, p = 0.245) or CPT (r = 0.099, p = 0.654) and total STAI scores for subjects with OA. There was a significant correlation between Global PPT and total WOMAC score (r = -0.381, p = 0.037) but no correlation between total WOMAC score and global CPT (r = 0.265, p = 0.11).

## Discussion

This study compared the mechanical and thermal pain thresholds of subjects with WOMAC-rated mild to moderate knee osteoarthritis with the thresholds of matched healthy controls. The results suggest that both widespread mechanical and cold hyperalgesia may be a feature of the pain experience for patients with OA of the knee.

### OA subjects showed widespread pressure hyperalgesia

Subjects with knee OA exhibited significantly lower pressure pain thresholds at the index knee compared with their matched healthy counterparts. This increased mechanical hyperalgesia local to an OA joint has been reported in a number previous studies [13–20] and is characteristic of localised sensitisation. The current study also found increased mechanical hyperalgesia distally and proximally to the OA joint, at both the ipsilateral ankle and elbow, with OA subjects showing a 20% decrease in pressure pain threshold across all sites. This pattern of widespread mechanical hyperalgesia also reflects the findings of a number of recent studies [13–20]. Imamura et al [14] reported significantly decreased PPT in the upper limb for subjects with knee OA. Neogi et al [37] reported significantly increased pain sensitivity across four upper limb sites in subjects with OA of the knee. Arendt-Nielsen at al [15] found decreased PPT at both the ipsilateral tibialis anterior muscle and extensor carpi radialis longus muscle in the forearm of subjects with knee OA, although neither were reported as significantly different to control subjects.

### OA subjects showed widespread cold, but not heat hyperalgesia

Subjects with knee OA also displayed significantly increased cold pain thresholds compared with pain free controls. As with pressure pain thresholds, CPTs were significantly elevated (more sensitised) both at the affected knee and also at the unaffected lower limb and upper limb sites. At each site, those with OA experienced their pain threshold at a temperature 40–47% higher than controls.

There is little available data with which to compare these results. Kosek & Ordeberg [38] reported significantly increased CPTs in subjects with hip OA prior to arthroplasty, with a mean CPT of 19.1°C, compared with 12.1°C for controls. In contrast, Fingleton et al. [20] report a lack of evidence for cold pain sensitivity in OA knee subjects. However, this review also points out that a meta-analysis for CPT was not possible due to limited data and varying methodology. Several of the more recent studies have used a cold pressor test rather than a threshold to pain test [17–19]. This test of pain tolerance may be more strongly influenced by external neuropsycholgical factors such as anxiety [39]. The current study applied CPT whose pain threshold methodology is similar to that for PPT and HPT and so meaningful comparisons are difficult to make. Additional comparative data with larger cohorts is clearly needed.

QST studies evaluating other musculoskeletal pathologies have also reported elevated CPT. For example, Sterling et al [8] found a mean CPT of 17–18°C in the 21% of whiplash subjects who showed poor recovery at 6 months and 2 years post injury. Cold hyperalgesia has also been reported for subjects with fibromyalgia (mean CPT 18.6°C) [6]. Given that cold hyperalgesia is considered to be an important prognostic indicator in patients with conditions such as whiplash associated disorder it is important to have data indicating that widespread cold hyperalgesia is a feature of patients with knee OA.

In contrast to PPT and CPT, no significant difference in mean HPT was found between subjects with OA and controls at any of the tested sites. Indeed, HPT values were remarkably consistent across sites for all subjects, with less than 1°C difference between maximum and minimum values. Although thermal hyperalgesia is described as a cardinal sign of neuropathic pain syndromes, there is ambivalent evidence about its role in musculoskeletal disorders. For example, similarly to the current study, Berglund et al [6] found that subjects with fibromylagia showed only cold but not heat hyperalgesia, as did Sterling et al [8] for patients with whiplash. Wright et al [3] showed no significant difference in HPT in patients with tennis elbow and Wylde et al [13] found no difference in HPT in subjects with knee OA. Although, King et al. [17] report a statistically significant difference between knee OA and control groups for HPT, the actual difference is once again less than 1°C. It therefore appears that heat hyperalgesia is much less common in patients with OA pain than cold hyperalgesia.

### Centrally driven hyperalgesia?

The finding that mechanical and cold pain thresholds are reduced at a range of sites throughout the body lends support to the notion that pain in osteoarthritis may be influenced by both local and central factors. A range of previous studies have supported this hypothesis of central sensitisation. Bajaj et al [24] demonstrated increased intensity and area of secondary hyperalgesia in OA subjects. Other studies have reported increased temporal summation [15] indicative of altered nociceptive system processing.

### Group differences in anxiety scores

Subjects with knee OA showed significantly higher levels of both state anxiety than control subjects although not higher trait anxiety. This partially supports a recent study by Ferreira et al. [40], which showed significantly higher levels of STAI state anxiety in OA compared with controls, although also reporting a significant difference in trait anxiety. It may be noted that this was a female-only sample in contrast to the gender-balanced current study, and that overall raw anxiety values were considerably higher. Although in the current study it might be argued that higher situational anxiety might be anticipated from a chronic pain cohort, the difference between studies emphases the challenge of determining whether neuropsychological factors are the cause, the effect, or unrelated to chronic pain.

### Study Limitations

The limitations of the current study must be acknowledged. In comparison to other QST studies, the sample size is relatively low and inevitably a community cohort of volunteer subjects will carry with it some selection bias. The amount of specific demographic and medical information collected on the participants was limited, as volunteers were screened and excluded if they fulfilled any of the exclusion criteria, including additional comorbidities such as fibromyalgia.

## Conclusions

However, within its limitations discussed above, the present study provides additional clear evidence that knee osteoarthritis is characterised by the presence of widespread hyperalgesia, in common with a number of other musculoskeletal disorders. Although the evidence for mechanical hyperalgesia is strong there are few previous studies which provide clear evidence of associated cold hyperalgesia in this patient cohort. This study therefore provides evidence of widespread multi-modality changes in nociceptive system function, thereby supporting the concept that knee osteoarthritis can no longer be seen as a purely peripheral pain disorder in all patients.
